# Recognising Consumers' Contributions to Health Research: Co‐Designing a Remuneration Framework for the Australian Context

**DOI:** 10.1111/hex.70314

**Published:** 2025-05-28

**Authors:** Mingming Zhou, Anne Parkinson, Lucy Clynes, Jane Desborough

**Affiliations:** ^1^ Department of Health Economics, Wellbeing and Society, National Centre for Epidemiology and Population Health (NCEPH) Australian National University (ANU) Canberra Australia; ^2^ Research Australia Canberra Australia

**Keywords:** consumer engagement, framework, health research, patient and public involvement, reimbursement, remuneration

## Abstract

**Background:**

Consumer engagement ensures research reflects lived health experiences and remains relevant to consumers' needs. However, challenges persist in appropriately recognising their contributions. Remuneration for consumers' time, skills and expertise, alongside reimbursing out‐of‐pocket expenses, remains a key challenge. While individual remuneration policies exist across Australian states and territories, a cohesive national framework has yet to be developed.

**Objective:**

This study aimed to develop a remuneration framework recognising consumers' contributions to health research in Australia.

**Methods:**

The framework was developed through: (1) a review of the international literature and Australian state‐ and territory‐based consumer advocacy organisation remuneration guidelines, (2) a stakeholder survey of current practices and (3) integration of findings to develop four preliminary remuneration models, which were presented for deliberation in six workshops with key stakeholders. Workshop data were analysed using framework analysis.

**Results:**

Workshop participants included twelve representatives from research organisations and nine consumers with chronic conditions. The final framework comprises: (1) decision‐making considerations, (2) remuneration rates (project‐based, meeting‐based and hourly rates), (3) payment methods and (4) non‐financial recognition approaches. The framework was designed to address the needs of diverse organisations, from small not‐for‐profit entities to larger research institutions with dedicated funding.

**Conclusion:**

This study developed the first nationwide remuneration framework to guide the Australian research community in recognising consumers' contributions to health research. While not the only model for remuneration decision‐making, it serves as a valuable starting point for dialogue and future policy development. Further evaluation incorporating consumer partners' experiences across diverse research contexts is essential to enhance the framework's applicability and foster fair recognition for contributions to research.

**Patient and Public Contribution:**

A project working group, including representatives from nine Research Australia member organisations, was actively involved throughout the study. These included three large universities from three different Australian states, one state‐based local health district representative and five national independent not‐for‐profit research organisations, including four consumer‐led disease‐specific organisations. Each organisation was represented by 1–2 people at each meeting. Including four members of the research team and two members of Research Australia, each meeting was comprised of 18–20 people. Their contributions included refining study aims, reviewing and piloting the survey, conceptualising remuneration models, recruiting workshop participants and finalising the remuneration framework. Input was gathered through three online meetings and email exchanges. The first meeting focused on survey design and distribution; the second on survey findings, remuneration models and recruitment strategies; and the final on reviewing workshop findings and co‐designing the remuneration framework. Additionally, 21 participants (9 consumers with lived experience of chronic conditions and 12 organisational representatives) contributed to six deliberative workshops.

## 1 Introduction

Consumer engagement in health research is increasingly recognised for its value in enhancing the relevance and quality of research [1, 2, 3]. Defined as individuals with lived experience of specific health conditions, consumers bring experiential insights into research and contribute to the design, conduct and dissemination of research, ensuring that outcomes align with real‐world needs [4, 5, 6]. While various terminology exists in the international literature, for the purposes of this study, ‘consumer’ is considered synonymous with related terms, including patient and public involvement and patient engagement. Despite growing acknowledgement of their expertise, the question of how to adequately recognise and remunerate consumers for their contributions remains a critical issue in health research [2].

Consumer engagement in health research can be categorised either by the role of the consumer or the level of engagement [7, 8, 9]. The role refers to the different activities that consumers become involved with in research, such as research participant, adviser, co‐researcher, consumer leader or recipient of knowledge [7, 8]. On the other hand, the level of engagement describes the degree to which consumers are actively involved in the research process [9]. For example, ‘inform’ involves providing consumers with information on engagement activities in health research, while ‘involve or engage’ entails direct, ongoing participation in specific research projects. ‘Consult’ typically includes activities such as offering feedback on research, whereas ‘collaborate or partner’ represents a deeper involvement, with consumers working alongside researchers as part of the team. For consistency, our study adopts the level of engagement to categorise consumer engagement, as outlined in Table 1.

**Table 1:** Levels of consumer engagement in health research.

Level of engagement	Description
Informing	Consumers are receivers of research information.
Participation	Consumers respond to surveys and take part in focus groups, interviews or deliberative workshops.
Consultation	Consumers sit on steering committees or advisory groups to provide guidance to the project team.
Collaboration	Consumers partner with researchers, as research team members, to co‐design research questions, review study protocols or help disseminate results.
Consumer‐leading	Consumers lead research projects.

Consumer remuneration practices vary considerably across international contexts. In some countries, remuneration has become a standard practice, supported by structured frameworks that outline how consumers should be remunerated for their involvement. In the United Kingdom, the National Institute for Health Research (NIHR) provides clear guidelines for remunerating consumers for their time and expertise [10]. Similarly, the Canadian Institutes of Health Research (CIHR) have established policies that emphasise fair compensation for consumers engaged in health research, offering guidelines on reasonable remuneration rates according to the type and duration of engagement [11, 12]. In contrast, many countries, including Australia, lack a unified approach to recognise consumers' contribution in health research. Notably, no national guidelines for consumer remuneration have been identified in Australia, creating inconsistency and inequity in recognition practices [13].

Remuneration practices in Australia are inconsistent and vary according to project budgets, funding policies and institutional practices [14, 15]. While research initiatives often reimburse expenses such as travel and accommodation, financial remuneration for consumers' time and expertise is less commonly provided. Results from a cross‐sectional survey of health research organisations in Australia found that while most organisations supported recognising consumer contributions to health research, only 56% offered financial remuneration and just 36% provided non‐financial recognition, such as training opportunities and acknowledgement in academic outputs [16]. This inconsistency not only impacts the equity of consumer participation across organisations but may also affect the inclusivity, diversity and ultimate quality of the research.

This gap in standardised remuneration practices points to a need for a cohesive framework that can guide research organisations in appropriately recognising consumer contributions. The aim of this study was to develop a remuneration framework to provide guidance for recognising consumers for their engagement throughout the research process. The specific objectives were to:

1. Review international and Australian existing guidelines for consumer remuneration,
2. Examine current consumer remuneration practices amongst Australian research organisations and
3. Co‐design a remuneration framework through consultation with research organisations and healthcare consumers.

## 2 Methods

### 2.1 Setting and Context

Research Australia, a national alliance of health and medical research organisations, collaborated with researchers from Research Australia to develop a remuneration framework supporting consumer engagement in health research in Australia. A multiphased, co‐design process was implemented to ensure the resulting framework was relevant and acceptable to health research organisations and consumers. Ethical approval was obtained from the Australian National University Human Research Ethics Committee (Protocol 2023/1263), and informed consent was secured from all participants before the survey and workshop phases.

#### 2.1.1 Working Group Formation

A project working group was established, comprising representatives from nine member organisations affiliated with Research Australia. These included three large universities from three different Australian states, one state‐based local health district representative and five national independent not‐for‐profit research organisations, including four consumer‐led disease‐specific organisations. Each organisation was represented by 1–2 people at each meeting. Including four members of the research team and two members of Research Australia, each meeting was comprised of 18–20 people. The working group remained actively engaged throughout the project via online meetings (*n* = 3) and email exchanges. Their responsibilities included refining the study's aims and objectives, providing feedback on survey design and piloting, conceptualising potential remuneration models for workshop discussions, recruiting workshop participants and reviewing and finalising the proposed remuneration framework.

### 2.2 Framework Development

**Figure 1:**
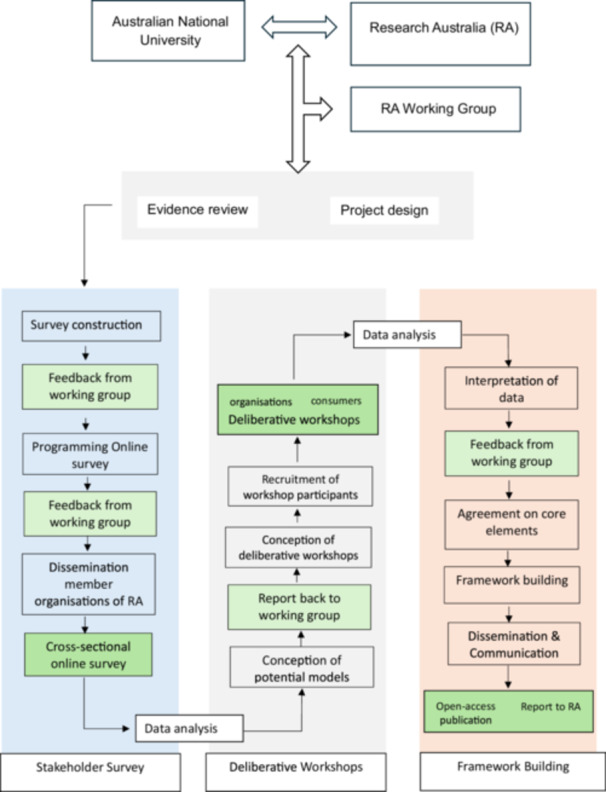
Project framework with indication of consumer engagement.

A three‐phase approach included: (1) an evidence review, (2) a stakeholder survey and (3) six deliberative workshops. Figure 1 outlines the framework development process, highlighting how and where the working group and key stakeholders were involved.

A sequential exploratory mixed‐methods approach was adopted to ensure that research questions, survey design and distribution, and the recognition models were iteratively developed and refined based on continuous feedback from the working group, research organisations and consumers.

#### 2.2.1 Evidence Review

The research team conducted an evidence review, including both academic sources and web searches, to identify existing frameworks and key components for recognising consumers' contributions to health research. The search strategy is presented in Table 2. The web search was conducted in two rounds. The first round applied similar search terms to identify grey literature not published in academic journals. The second round combined ‘consumer engagement’ and ‘consumer and community involvement’ with the names of Australia's eight states and territories to locate relevant policy documents from consumer organisations. All identified sources were reviewed to extract approaches for recognising consumers' contributions within the context of consumer engagement.

**Table 2:** Search strategy for the evidence review.

	Academic sources	Web sources
Search databases	Scopus and PubMed	Google
Time scope	Last 10 years	
Search terms	“patient partner”, “consumer partner”, “consumer engagement”, “consumer and community involvement”, “patient engagement”, “public and patient involvement”, “patient partnership”, “research”, “remuneration”, “compensation”, “payment”, “reimbursement”.	1. Similar terms with the academic search
		2. Combined “consumer engagement” and “consumer and community involvement” with the names of Australia's eight states and territories to locate relevant policy documents from consumer organisations
	Example search: PubMed: ((“patient partner”[Title/Abstract] OR “consumer partner”[Title/Abstract] OR “consumer engagement”[Title/Abstract] OR “consumer and community involvement”[Title/Abstract] OR “patient engagement”[Title/Abstract] OR “public and patient involvement”[Title/Abstract] OR “patient partnership”[Title/Abstract]) AND “research”[Title/Abstract] AND (“compensate*“[Title/Abstract] OR “remunerat*“[Title/Abstract] OR “pay”[Title/Abstract] OR “reimburse*“[Title/Abstract])) AND (y_10[Filter])	Example search: Google: “consumer engagement”, “consumer and community involvement”, South Australia

#### 2.2.2 Stakeholder Survey

This phase examined how health organisations (both for‐profit and not‐for‐profit) recognise and remunerate consumers for their contributions to health research. The objective was to explore health organisation members' experiences and attitudes toward recognising consumers.

A cross‐sectional survey was developed by the research team in collaboration with the working group, drawing on their knowledge and experiences in remunerating and recognising consumers. The survey consisted of 67 multi‐choice and open‐text questions and was comprised of five parts: (1) basic information about the participating organisation, (2) forms of recognition of consumer engagement, (3) forms of financial remuneration and reimbursement, (4) remuneration processes and (5) perspectives about remuneration guidelines.

The survey was distributed to all Research Australia's member organisations (approximately 160) and remained open for 10 weeks (10 December 2023 to 29 February 2024). Details of the survey development process have been reported elsewhere [16].

#### 2.2.3 Deliberative Workshops

Deliberative workshops were selected as the most appropriate method for developing the remuneration framework, given their ability to support nuanced discussions of complex ethical and practical considerations [17]. This approach enabled diverse stakeholders, including research organisation representatives and consumers with lived experience, to collectively examine, critique and refine preliminary remuneration models informed by the evidence review and survey findings. The deliberative format encouraged deep reflection, exploration of potential implementation challenges and the development of informed, context‐sensitive recommendations.

Findings from the first two phases informed the development of potential models for recognising consumer contributions to health research for deliberation in the workshops. These were first presented and discussed with the working group in an online meeting before being introduced to participants in deliberative workshops to assess their feasibility and acceptability. Separate workshops were held for research organisations and consumers.

The workshops followed a two‐stage structure. In the first stage, researchers introduced each remuneration model, and in the second stage, participants provided feedback on the feasibility and acceptability of key elements, such as financial remuneration methods and rates. The workshops were facilitated by M.Z. and J.D.

### 2.3 Collaboration With the Working Group

Findings from the evidence review, stakeholder survey and deliberative workshops were reviewed and discussed with the working group across three online meetings. Meeting discussion contents can be found in Table 3.

**Table 3:** Discussion contents for the working group meeting.

	Discussion contents
First meeting	The draft survey and participant information sheet were shared for feedback, and decisions were made regarding the survey distribution process.
Second meeting	Discussion focused on survey results and potential remuneration models for the deliberative workshops, including model structure, key elements and the workshop recruitment strategy. Additional feedback was gathered via email between meetings.
Third meeting	Results from the deliberative workshops and the proposed remuneration models were presented. The group discussed and finalised the models, which were then mapped onto a four‐level recognition framework developed with the working group to illustrate the different methods for recognising consumers' contributions to health research.

### 2.4 Data Analysis

A sequential approach was used, with quantitative and qualitative data from the stakeholder survey analysed first to inform the deliberative workshops. M.Z. conducted the analysis, with regular review and discussion with J.D. and A.P.

#### 2.4.1 Stakeholder Survey Analysis

Descriptive analysis was used for multiple‐choice questions, while content analysis was applied to explore open‐text responses. Descriptive statistics were conducted using IBM SPSS Statistics 29, and data from open‐text fields were imported into NVivo 14 (QSR International) software for storage and analysis.

#### 2.4.2 Deliberative Workshops Analysis

Data collected from the deliberative workshops were transcribed and analysed using framework analysis. Developed specifically for applied policy research, this method was selected to provide a systematic and transparent method for analysing feedback on the proposed remuneration models, enabling targeted insights that could be directly applied to policy and practice [18, 19]. Its comparative structure facilitated efficient cross‐sectional examination of perspectives from both research organisations and consumers, allowing us to identify areas of consensus and divergence regarding remuneration practices. Additionally, the approach's robust capacity to manage complex qualitative datasets from multiple stakeholders made it particularly valuable for synthesising the diverse viewpoints expressed during our deliberative workshops [19].

## 3 Results

Over the course of 1 year, the working group met online quarterly to guide the model development.

### 3.1 Phase 1: Evidence Review

We screened 214 articles identified by the literature searches (Scopus and PubMed) and 37 documents from web searches (Google). After title and abstract screening, 21 documents were reviewed in full text. Among them, we identified a scoping review published in December 2023 that summarised guidance and policy documents on consumer compensation, aligning well with the purpose of this study [13]. Due to the comprehensive nature of this scoping review, it was adopted as an international reference. It synthesised non‐financial and financial compensation methods from 65 identified documents, providing a thorough overview of financial remuneration recommendations across three categories: consultation, collaboration and other forms of engagement in research activities. Remuneration methods currently recommended by Australian state‐based consumer organisations were charted in Table S1.

Additional documents related to the recognition of consumer engagement were also retrieved, including from the National Institute for Health and Care Research (NIHR) in the United Kingdom, the Patient‐Centred Outcomes Research Institute (PCORI) in the United States and the CIHR [10, 11, 12, 20].

### 3.2 Phase 2: Stakeholder Survey

A survey was developed based on the evidence review and feedback from working group members and distributed to Research Australia member organisations. A total of 110 participants completed the survey, and their responses were included in the analysis. More than 80% of respondents were from the academic field (47% from universities, 29% from medical research institutes and 10% from research organisations). The remaining participants represented healthcare providers, non‐government organisations, state government bodies, research consultancies and community health groups. Most organisations advocated for recognising consumer contributions through both financial and non‐financial means; however, there was variability in current remuneration practices, methods and rates. Despite this, the survey findings indicated strong support from participating organisations for the establishment of national guidelines or recommendations for consumer remuneration practices. Detailed participant characteristics and survey results have been presented elsewhere [16].

### 3.3 Phase 3: Deliberative Workshops

Integration of the results from Phases 1 and 2 informed the development of four potential models that addressed key aspects of consumer remuneration: remuneration rates and methods, non‐financial recognition methods and a decision‐making support tool for recognition methods.

#### 3.3.1 Stage 1: Model Development

Based on the survey results (Table S2), the majority of engagement activities (85.2%) focused on collaboration, consultation and participation. Similarly, recommended monetary values for remuneration in international contexts centred on these three levels of engagement [13]. While from the remuneration methods recommended by state‐based organisations in Australia, only the Health Consumers' Organisation in Victoria provided specific rates for consumer‐led engagement (Table S1). Consequently, the first remuneration model outlined rates and ranges for collaboration, consultation and participation (Table S3).

The second model addressed remuneration methods and reimbursement coverage, which was developed based on survey results and the evidence review (Table S4). Methods of remuneration and reimbursement, or non‐financial methods of recognition, were not mentioned in the Australian state‐ and territory‐based recommendations. The third model focused on non‐financial recognition methods. Twelve non‐financial recognition methods were outlined (Table S5), including those identified in the review and reported by survey participants, highlighting both similarities and differences. The fourth model was a decision support tool for remuneration identified in the literature [21]. It was presented in the workshops to consult stakeholders on its potential application within the Australian context.

The four potential remuneration models were presented to the working group for feedback, and during this online meeting, participants confirmed the four models and provided suggestions on the recruitment strategy for deliberative workshop participants.

#### 3.3.2 Stage 2: Conduct of Deliberative Workshops

Six deliberative workshops were conducted: three for consumers and three for research organisations. A total of 21 participants were recruited via mailing lists of Research Australia members and through the research team's networks. Participants included nine consumers and twelve organisational representatives (see Table 4). The meetings were lasted for 60–90 min. Audio recordings from the workshops were transcribed, and framework analysis was conducted following Laurie J. Goldsmith's five‐step approach [19]. The results are presented according to the four potential models developed in Stage 1: (1) remuneration rates, (2) remuneration methods and reimbursement coverage, (3) non‐financial recognition approaches and (4) utility of the remuneration decision‐making support tool. Selected illustrative quotes from the workshops are provided in Tables 5, 6, 7, 8, organised under these headings.

**Table 4:** Participants characteristics of deliberative workshops.

No. of workshops	No. of participants		Gender	
	Organisational representatives	Consumers with chronic conditions	Male	Female
1	2			2
2		3	3	
3		2		2
4	5		1	4
5	5		1	4
6		4		4
In total	12	9	5	16

**Table 5:** Rates of remuneration: illustrative quotes from workshops with consumers with chronic conditions and member organisations of Research Australia.

	Illustrative quotes from participants	
Methods of rates	Member organisation from research Australia	Consumers with chronic conditions
Flat rate	I think in a participation sense, having a flat rate, if you do this, then you get a $20 gift card or something like that. A flat rate, I think, is something much more acceptable as opposed to any kind of hourly rate. (M1) In this category, I like the idea more of a one‐off amount and a flat rate…. The researcher would have to be very clear on the expectation and stick to the time limit. (M1) I would be more inclined to put a flat rate and then people can go up or down, just because I know we all will be trying to make savings everywhere. It also values the input from the consumer, like it's saying this is how important we believe it is and then it takes into consideration all those other variables and issues which come out. (M4)	I think you'd want to limit the administrative burden on the people paying so pro rating hours and things is a bit of an annoying thing. Perhaps in that context, maybe some lump sum might be easier to manage. (C6) I think just a flat rate, which is enough to be a recognition, but not enough to bankrupt the research project and also not enough to make it difficult for administrators. (C6)
Meeting rate	I find the hourly thing a bit tricky. I think we prefer generally to talk about it as a meeting, and then it can encapsulate if the meeting goes 15 min longer or something. If people are really counting, then that allows a bit of variability in the length of the meeting, but also you can say you get offered $100 per meeting, and that includes any prep for the meeting. You're not measuring what that prep is, but I guess in the sense that a person can then decide if they do a lot of prep before a meeting or not, depending on what they're able to do. I think it's a bit cleaner to have a meeting. (M1) If you've got like pay per meeting, but that meeting includes the prep like you said. You just bulk it all up together…. It's just like you've got to take into account some people might take longer to do things. (M4)	I don't know how other guys think about it, but that's where I'm at. Probably per meeting because there's always going to be—there tends to be more work before or after meetings that might go unaccounted for. (C2)
Hourly rate	I just briefly heard the conversation though about remunerating people for not only the meetings that they attend but for any preparation work that may need to be done before the meeting or any follow‐up work after the meeting, in recognition of people who are involved as consumers may be working in other roles, and may not be able to do their main role during that time. I think a fair remuneration for the hours required is important to consider. (M4)	
Rates amount		
Participation: $50/h	I think for something like an interview focus group (*participation*), $50 is what we tend to offer…. In this category, I like the idea more of a one‐off amount and a flat rate. I think $50 is reasonable. (M1)	I think maybe one way of looking at it might be to look at the minimum wage in Australia, hourly rate in Australia and maybe then double it. That might be one way of doing it and then at least you've got a way of calculating it. Actually, the minimum wage is $24.10 per hour. If we double it, that's $50 an hour. (C3) We're just saying if you had to come up with an amount that you thought was a rip for organisations who have the money to pay, what would you consider a fair and reasonable amount? I'd consider $50 (per hour) to be reasonable. (C6)
Collaboration: $40–50/h		I think as a team member (*collaboration*), my experience has been typically between $40 and $50 an hour for a team member, and then slightly higher for a chairperson. (C2)
Consultation: Varied depending on the project	I think for something like an interview focus group, $50 is what we tend to offer, whereas I think when you've got the team member in *advisory roles*, I think a flat rate can work, but it needs to be *a bit more* because there's an assumption that there's a bit of in‐between stuff as opposed to an isolated activity. (M1) For the team members in the *advisory role*, because it could be on a more ongoing basis, that *lower rate* more consistently is more appetible than for the active participants. (M4) The team members advisory roles, yes, I get it because I think that if we're going to really legitimise this, if they truly are team members or their advisory roles which is a subversion of a team member, they should be seen as any other, say research assistant that's working on the project. That's how we build these *salaries in and pay them*. I don't think that's inappropriate. (M4)	
Payment implications		
Tax impact	From a tax perspective you might be talking about fringe benefits. As a not‐for‐profit, we're exempt from fringe benefits but that may not be the case for everyone. It definitely shows up in our bank account. We're audited every year so that's how we manage those payments. To be honest then, it's not a lot of money. We've been involved in three or four research projects and it's hundreds of dollars, not anything more than that. (M4) Because it's an honoraria, then they don't need to‐‐ Yes, so they understand that it's not part of their taxable income. It's an honoraria, yes. That is a concern for some people who might have that conversation. (M1)	At the moment, I have to take into account my larger committees into my taxation considerations. Come November, when I become eligible for part pension, I'll be limited by how much income I can earn across those committees. I may have to withdraw from some so as not to reduce my pension. (C2)
Pension impact		At the moment, I have to take into account my larger committees into my taxation considerations. Come November, when I become eligible for part pension, I'll be limited by how much income I can earn across those committees. I may have to withdraw from some so as not to reduce my pension. (C2)

*Note:* Deliberative workshops with consumers with chronic conditions (C). Deliberative workshops with member organisations of Research Australia (M).

**Table 6:** Remuneration payment methods and reimbursement coverage: illustrative quotes from workshops with consumers with chronic conditions and member organisations of Research Australia.

		Illustrative quotes
Payment methods	Member organisation from research Australia	Consumers with chronic conditions
Gift card	There are others who would prefer gift cards because I guess with gift cards, they're easier to spend, rather than having them go in and out of their bank account. They don't necessarily have to declare that income, if it doesn't go in and out of the bank account, so it doesn't affect them from a tax perspective. (M4) One of the big problems we have is that the administrative burden around paying someone for a contribution like that by putting them on the payroll and setting up super is usually out of proportion with the service they're providing to us. Highly reluctant to do that. It's usually an honorarium arrangement paid through a gift card type or a prepaid visa. (M5) I think avoiding any issues with benefits or tax issues, I think gift cards are probably my preference. It just cuts out all that issue. (M5)	I think my experience, again, it's either been direct transfers or gift cards. They've been the basic choices; I would certainly steer away from recommending cash. (C2)
Bank account transfer (honoraria)		Most of mine has been by bank account, but a larger number or smaller amounts were by gift cards. If it's less than a certain amount, it's easier to receive a gift card. If it's an ongoing involvement with regular meetings, I've used the bank account method. (C2) I'm just coming from a consumer perspective. We're often asked to be involved in consumer feedback or research, and we've been remunerated in a number of ways. Gift cards is one and an hourly rate or a rate per meeting is the other. From our perspective either way is okay with us. The not‐for‐profit, as long as everything's agreed upon and contextually suitable, we probably prefer a cash payment or an honorarium or something that goes into our bank account. (M4)
Prepaid Visa or Master card	…the prepaid Visa or MasterCard are preferable to the gift cards, which I think often have a broad number of places you can spend it, but then can still be limiting for some people, particularly people who live in rural and regional areas, and that having basically the equivalent of cash, which is the prepaid Visas or MasterCard is their preference and then they can spend it where they wish. (M1)	I would lean towards what P2 (another consumer participant) recommended where you have the option, but it either be bank account or the prepaid cards or the gift cards. The flexibility of the prepaid cards is always great. (C2)
Cash		I think my experience, again, it's either been direct transfers or gift cards. They've been the basic choices; I would certainly steer away from recommending cash. (C2) Well, give me the cash, thanks. I think that's an obsolete one for Australia, isn't it? It's becoming so much more difficult to even get cash. I've never received cash, actual cash. (C3) I'm just coming from a consumer perspective. We're often asked to be involved in consumer feedback or research and we've been remunerated in a number of ways. Gift cards is one and an hourly rate or a rate per meeting is the other. From our perspective either way is okay with us. The not‐for‐profit, as long as everything's agreed upon and contractually suitable, we probably prefer a cash payment or an honorarium or something that goes into our bank account. (M4)
Similar to employees	The team members advisory roles, yes, I get it because I think that if we're going to really legitimise this, if they truly are team members or their advisory roles which is a subversion of a team member, they should be seen as any other, say research assistant that's working on the project. That's how we build these salaries in and pay them. I don't think that's inappropriate. (M4)	
Reimbursement coverage		
All out‐of‐pocket costs	We would cover any out‐of‐pocket costs, including, particularly if it's a person living with dementia, paying for their carer to come, whether that's attend the conference, if it's a conference, or any transport and things like that. Then any food or whatever if they're away from home. (M1)	I think with these, they should be covered at cost. I don't think that's, especially if you're attending in person, it's not much, it's not much of a question to me, but I don't know. That's what I would think. (C2) Anyone out‐of‐profit cost, I think need to be covered. I look at childcare and care is tricky, but if you want someone to be at a conference or a meeting and they have to pay for child care, I guess that that should be considered as a reimbursement. (C3)
o IT and phone charges	I probably, just picking up on the IT and phone charges, definitely support that because if they're expected to take phone calls and that as well, but it's also, I guess, that personal use of devices as well if they're going to meetings. I think it's good that's been picked up there as well. (M4)	
o Support worker or a carer		If you've got to have a support worker or a carer, so maybe you need to expand the word carer to include a support worker, because they might determine a support worker as being a carer. If you've got to pay an extra fee for the support worker, you should make sure they're covered as well. (C2)
Being upfront		
o Pay up front	What I recommend to researchers where possible if you are covering a taxi for an advocate to come to and from a meeting or a seminar or whatever, to post the cab charge vouchers out to them beforehand, so then that's covered. Then, no one has to hand in receipts or anything like that. I agree with P2, it can feel—Particularly if it's parking and it's maybe a few dollars, it's not really worth it. If they're doing that all the time, then it is worth it. Even if it is a few dollars, it's worth it. (M1)	
o Notice up front	It's being upfront with the researchers as well, because then the researchers will need to determine whether they actually have the funds to provide that level of reimbursement. If they don't know those things in advance, and then the person with lived experience just sends them all these bills, then the researcher might get a bit of a shock and go, ‘Oh, shit. I didn't know I had to pay for the carer as well. That's double the costs.’ (M1) I think just being upfront with people because I think most advocates or consumers would understand the limitations of people's funding. Just presenting them with the opportunities and the information, and saying, ‘This is what I can manage. I didn't want to be involved.’ In the same way that if a project runs way over time, but there's no money left, saying to people, ‘We can't pay you anymore to do these last few meetings. There's no money. You can step off the working group or the advisory panel.’ Just giving people all the information they can just make the decisions themselves. I think it can be tricky, though because facts when money is the limiting factor for something to happen for someone, but that's the reality, isn't it? (M1)	

*Note:* Deliberative workshops with consumers with chronic conditions (C). Deliberative workshops with member organisations of Research Australia (M).

**Table 7:** Non‐financial recognition methods: illustrative quotes from workshops with consumers with chronic conditions and member organisations of Research Australia.

	Illustrative quotes	
	Member organisation from research Australia	Consumers with chronic conditions
Co‐authorship/acknowledgement on academic outputs	I think things particularly like co‐presentations at conferences and even co‐authorship and things like that, for some advocates or consumers that's really nice. Authorship on a paper to a consumer really means the same thing as authorship on a paper to an academic, which is like a KPI, or a presentation at a conference. (M1)	I found co‐authorship and co‐presentation to be two of the most rewarding parts of the process of participating on these sorts of projects. Yes, I would definitely recommend that they be, if that is a possibility, if there are publications that come out of a piece of research, then our name should definitely be on it if it's possible. (C2) I think that's a very good idea to have your name on a publication right at the end (acknowledgement). It makes you feel you have done something towards that. It's a very tangible exercise for them. They can see the difference they're making. It makes a huge difference, I think. (C6)
Co‐presentations at conferences	I think from a co‐presentation perspective at conferences we've done a lot of that with universities and it provides a good alternative perspective in our case from a patient view. We've presented at academic and healthcare professional conferences with our research partners, and I think that's a really great recognition method and it also solidifies the partnership. That's something that has worked really well for us and I think it's an important non‐financial recognition. (M4) We had our conference last year in Perth, which would hold every two years and we had quite a few consumers attend the conference, but we also had a researcher from the Menzies Institute with his consumer buddy there with him presenting with him. It was such a beautiful presentation just to see that partnership. I think everyone appreciated that, because we had a day dedicated to how to involve consumers in research, not that we know 100%, but just to see how others are doing it. I think co‐presenting is quite a nice thing to do. (M4)	I think the academic recognition is really valuable and worthwhile. That's something I'd appreciate, I think. Maybe co‐presentation, none of the other things particularly. (C6)
Opportunities for skills development	I like the idea of skills development. That's really cool. I wouldn't have even considered that to be perfectly honest, so that's great. (M4)	In terms of training or opportunities for skills development, that's certainly an interesting one I haven't really considered before. If someone is keen to get more involved in research, especially people who might have that extra time, it's definitely something interesting. (C2)
Team building		I always say in addition to any paid remuneration, so if you've got a team building activity at the end of the major project, then all members of that team, be it researchers or consumers or others, should be involved because they are part of the team. (C2)
Other suggestions		
Public recognition	The other thing I was going to mention is just public recognition that we do. Getting names of research committees or advisory committees for projects on our website, naming them as consumer participants, and believe it or not, just certificates of recognition at the end. We sort of see them used, particularly with young volunteers for job applications and those sorts of areas. (M5)	
Consumer mentorship	The only other thing I thought, which I don't know if it really falls under non‐financial recognition, but maybe it's more in the support area, but I guess, buddying with other consumers or having that consumer mentorship as well. I know WAHTN has some sort of buddy system, but obviously, recognising that could be intimidating to be the only consumer or the group, or even if there's more than one, not having an experienced person to role model and show you that you can actually speak up and question things. (M5)	
Referrals to relevant projects		I think something else that is relevant and doesn't appear in this table is being referred on to others through the academic network as someone who's got something to offer and is not just seat warming. Part of the reason that comes to mind is that many people get thrown off the bike of their own careers by their own illness or the illness of children and parents. It's a bitter pill to swallow. Recognition that people are not dead from the neck up is really helpful. (C3)

*Note:* Deliberative workshops with consumers with chronic conditions (C). Deliberative workshops with member organisations of Research Australia (M).

**Table 8:** Decision tool to support remuneration decision‐making: illustrative quotes from workshops with consumers with chronic conditions and member organisations of Research Australia.

	
Researchers	Illustrative quotes
Application context	I think in some situations where you have an organisation or a group where potentially they don't have a lot of funding or minimal, or they're deciding whether they actually can even offer it, or should they. I think it could be potentially helpful for groups in that situation. Potentially for researchers who have never engaged with consumers before. Actually, some of these questions get you thinking about how you might plan your engagement activity. It gets them thinking about, ‘Well, okay, how many hours do I actually need this person for? Who are they going to meet with? Am I incorporating diversity?’ It actually gets them thinking about some important aspects of engagement. It's useful in that respect as well. (M1)
Add‐on to the tool: relevant skills consumers bring to research	I think one of the things that you probably need to consider on that tool is the relevance of the consumers or the skills that the individual or organisation brings to the research. That might sound like a very obvious thing to consider, but obviously, you need the right people in terms of consumer engagement. You've got equity there, which I think is important and vulnerable populations, but I think relevance or appropriate skills and knowledge could be another thing that you could add to that consideration. (M4)
Problematic scoring	I think the scoring might be problematic. I don't see it as being a practical tool to use. (M5)
Consumers	
Not relevant/useful to consumers	It might be more an organisational point of thing. From our point of view, we should be reimbursed or remunerated for our involvement. If you're going to get a research proposal up, such as if you went through the MRC, you've got to show your consumer involvement. This is just detail that I don't think we need to see. (C2) My initial reaction is not useful, overwhelming and too subjective. You'll be spending time justifying why you got paid and why you didn't. (C6)
Adding administrative burden	So much today, everybody thinks they can improve things. Improve things means coming up with something like this, which takes time and an administrative burden. Then, it wears people out. (C6)
Subjective nature	Quite divisive. Yes. Too subjective. It's almost as if our salaries were organised that way. You know what I mean? We all had a slightly different salary because— (C6)

*Note:* Deliberative workshops with consumers with chronic conditions (C). Deliberative workshops with member organisations of Research Australia (M).

##### 3.3.2.1 Remuneration Rates

This component explores remuneration rates across three levels of engagement: consultation, collaboration and active participation. Three subcomponents emerged: (1) approaches for determining rates, (2) recommended rate amounts and (3) payment implications. Supporting quotes from participants are available in Table 5.

(1) Participants discussed various payment approaches, including flat rates, meeting rates and hourly rates. Flat rates (set amounts/lump sums) were favoured by some participants for several reasons: firstly, they provide flexibility and practicality, allowing researchers to establish clear expectations while giving consumers autonomy in time allocation; then they reduce administrative burdens compared to tracking and calculating hourly payments; lastly, they benefit both researchers by minimising financial strain on projects and consumers by offering meaningful recognition. Participants emphasised the importance of researchers clearly communicating expectations and time commitments when implementing flat rates. While acknowledging potential variability in consumer contributions, participants generally agreed that a reasonable flat rate served as appropriate recognition without compromising research budgets.

(2) In terms of specific hourly or meeting rates, participants provided recommendations after considering the model presented during workshops (Table S3). For active participation (e.g., focus groups or interviews), most participants agreed that AUD $50/h was suitable, based on doubling Australia's minimum wage (AUD $24.10/h) [22]. For collaborative roles involving teamwork, participants recommended AUD $40–50/h. For consultation roles, opinions varied more significantly. One participant advocated for higher rates (> AUD $50 per hour) for consultation roles, while another suggested lower rates for these roles due to their ongoing nature. Another participant proposed that consumers in consultation roles be paid similarly to research assistants working on the project.

(3) Both research organisations and consumer participants highlighted important payment implications, particularly regarding taxation and government benefits. Representatives from small, not‐for‐profit (disease advocacy) research organisations expressed a preference for honoraria, which are classified as honorary rewards for voluntary services rather than fees for professional expertise. According to the Australian Taxation Office, these are not considered assessable income [23]. Consumer participants noted that financial payments received through engagement activities must be factored into their overall income reporting if they receive disability or age pensions. One consumer shared that she might consider withdrawing from certain committees to avoid earning amounts that could affect her eligibility for pension benefits.

##### 3.3.2.2 Remuneration Methods and Reimbursement Coverage

Participants discussed several methods for remuneration, including gift cards, bank account transfers, prepaid Visa or MasterCard, cash and employee‐style payments. Gift cards were favoured for their convenience and tax implications: the ease of use, lower administrative burden, and no need to be declared for tax purposes. Bank account transfers (direct deposit) were preferred for larger amounts or ongoing payments. Representatives from not‐for‐profit organisations noted that bank transfers were most appropriate when contextually suitable, particularly for substantial or recurring payments. Prepaid Visa or MasterCard options received positive feedback, with many participants preferring them over traditional gift cards due to their wider acceptance and greater flexibility in purchasing options. Participants appreciated that these cards could be used at most locations accepting card payments, unlike store‐specific gift cards. Participants' opinions regarding cash payments were mixed. Some preferred to avoid cash transactions entirely, while others—including consumer participants and not‐for‐profit organisation representatives—welcomed this option for its immediacy and simplicity. One organisation representative suggested treating consumers engaged in research as employees (similar to research assistants) and providing them with salaries, formalising the relationship and potentially providing additional benefits. Based on workshop feedback and team members' prior experiences, an ‘other’ option was added to the possible remuneration methods to accommodate consumers' requests that their remuneration be donated to a charity instead of receiving personal payment. Overall, participants suggested that researchers should provide consumers with multiple payment options when possible, allowing individuals to select the method that best suits their personal circumstances and needs.

All participants unanimously agreed that research projects should reimburse any out‐of‐pocket expenses incurred by consumers during their participation. Several specific expense categories were highlighted: organisation representatives emphasised the importance of covering technology‐related costs, such as IT equipment and phone charges, as consumers are frequently expected to engage in research activities via phone or the internet. Consumer participants suggested that support worker or carer fees should also be reimbursed when their assistance is necessary for participation. The need for upfront clarity about reimbursement coverage was emphasised, which involved two essential aspects: (1) Pay upfront: This approach involves reimbursing consumers for anticipated out‐of‐pocket expenses (such as taxi or parking fees) before they incur the costs, preventing consumers from having to pay expenses themselves and then seek reimbursement later, which can create financial strain. (2) Notify upfront: This principle applies to both parties—consumers should inform researchers in advance if they expect reimbursement for unforeseen costs related to their participation, such as support workers or carers. Researchers must be transparent about funding availability, acknowledge any funding limitations and offer alternative engagement options when full reimbursement isn't possible. These practices would ensure financial transparency and prevent misunderstandings about expense coverage. Supporting quotes from participants regarding reimbursement practices are available in Table 6.

##### 3.3.2.3 Non‐Financial Recognition Approaches

The discussion of non‐financial recognition approaches, informed by both international contexts and survey results, centred on four primary aspects (co‐authorship and acknowledgement on publications, co‐presentations at conferences, opportunities for skills development and team building), with three additional suggestions emerging from participant feedback (referrals to relevant projects, consumer mentorship and public recognition).

Most participants valued having their names included in research publications, either as co‐authors or through acknowledgements. This form of recognition provided tangible evidence of their contributions and was widely appreciated. Participants similarly valued opportunities to co‐present research findings at conferences. This approach was seen as strengthening the partnership between consumers and researchers while providing public recognition of the collaborative effort. While participants acknowledged the value of skills development opportunities such as training programmes, many noted they had not previously considered their significance. These opportunities were recognised as beneficial for both current and future engagement activities. One consumer participant emphasised the importance of team‐building activities, highlighting that they foster a sense of belonging, helping consumers see themselves as integral members of the research team rather than external contributors.

Beyond these primary recognition methods, participants proposed three additional approaches that were incorporated into the final framework under the ‘other’ option. (1) Project Referrals: One consumer participant advocated for being referred to other relevant projects through academic networks. This was particularly valued by consumers with chronic conditions who have reduced working capacity but can contribute meaningfully to research projects. (2) Consumer Mentorship: An organisational representative suggested implementing mentorship programmes where experienced consumers can guide newcomers, helping them to engage more effectively in research while recognising the expertise of experienced consumer partners. (3) Public Recognition: Participants highlighted the value of public recognition, such as listing engaged consumers on project websites. This was particularly valuable for younger consumers who use it as evidence of experience for job applications. Supporting quotes from participants regarding non‐financial recognition approaches are available in Table 7.

##### 3.3.2.4 Remuneration Decision‐Making Support Tool

The proposed decision‐support tool [21] generated divergent perspectives among workshop participants. Organisational representatives in two of three workshops (*n* = 7) viewed the tool favourably, recognising its potential to promote thoughtful planning of engagement strategies and optimise consumer contributions within research frameworks. They saw value in having structured guidance for considering engagement activities and their associated remuneration. However, participants in the third organisational workshop (*n* = 5) expressed the view that, from the lens of a consumer, one would believe that remuneration should not be contingent on how much a person contributes; rather, it should be a fundamental principle. Similarly, several consumers expressed scepticism toward the tool, characterising it as subjective. They considered such a tool unnecessary, arguing that consumer remuneration should be a fundamental principle and standard practice, rather than a decision requiring special deliberation. Organisational representatives suggested incorporating additional criteria into any remuneration tool, such as assessing the relevance of consumers or the specific skills that individuals or organisations bring to the research. These additions would help ensure that researchers engage the most suitable consumers for each project.

We considered this feedback in relation to the survey results, for which 92% supported consumer recognition and only 3% did not [16]. We also examined the available literature regarding the use of the tool, and while three articles acknowledged its value and one study protocol proposed its use for budget planning, no evidence demonstrated its implementation in actual remuneration practices [24, 25, 26].

After careful consideration of these integrated findings, the consensus was to exclude the tool and follow the suggestion to incorporate other criteria into the framework as suggested by workshop participants. We developed the following four key questions to guide remuneration decision‐making:

*(1) What forms of recognition will be offered to consumers participating in this project?*

*(2) What expenses are eligible for reimbursement under this project?*

*(3) Which forms of recognition do engaged consumers prefer?*

*(4) What potential financial implications should consumers consider when opting for financial recognition?*

We believed these questions provided a more flexible framework that acknowledged the fundamental importance of remuneration while allowing for contextual considerations. Supporting quotes from participants regarding the remuneration decision‐making tool are available in Table 8.

### 3.4 Framework Finalisation

Analysis of transcripts from the six deliberative workshops informed the development of two remuneration frameworks (Figures S1 and S2): one for funded research organisations and another for organisations without funded research. These frameworks and the evidence that informed their development were discussed in a meeting with the working group, where participants endorsed their core elements as essential for recognising consumers' contributions and suggested refinements.

A key suggestion was to integrate the two frameworks into a single one, ensuring non‐financial recognition for all consumers even when financial remuneration is possible and emphasising that non‐financial recognition cannot replace financial remuneration. Consequently, the final remuneration framework prioritises flexibility by offering three payment options (project rate, meeting rate and hourly rate) rather than prescribing a single approach. This flexibility extends to payment methods, allowing organisations to choose from options such as gift cards, bank transfers or prepaid cards based on consumers' preferences and circumstances. The framework also emphasises inclusivity and addresses equity issues by promoting consistent remuneration practices, regardless of project type or geographic location. This approach aims to encourage participation from consumers with diverse socio‐economic backgrounds, as they can anticipate fair remuneration for their valuable contributions, thus reducing financial barriers to engagement that disproportionately affect underrepresented groups.

**Figure 2:**
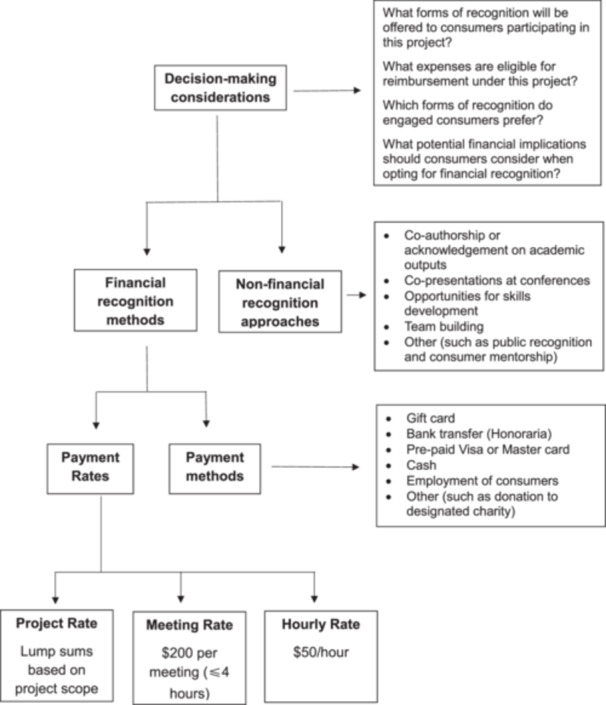
Framework to support financial and non‐financial recognition of consumers' contribution to health research.

Incorporating this feedback, the finalised framework (Figure 2) provides practical guidance to support financial and non‐financial recognition of consumers' contribution to health research based on funding availability and consumer preferences. The process begins with decision‐making considerations, where researchers evaluate available recognition options, including the availability of funding for remuneration and reimbursement, the implications of receiving payments for individual consumers, and discussions with consumers to identify their preferred methods of payment. The framework establishes that non‐financial recognition involves tailoring approaches to consumers' needs, while financial recognition requires discussions about payment methods and rates, ensuring all contributions are appropriately acknowledged regardless of circumstances.

## 4 Discussion

This study aimed to develop a remuneration framework to guide researchers in exploring recognition options and ensuring fair and consistent remuneration for consumer engagement in health research. Addressing a significant gap in the Australian research context, our framework supports informed decision‐making and policy formulation. Developed collaboratively with a working group of research organisations and consumers and informed by a review of national and international recommendations, a survey of current Australian practices and deliberative workshops with key stakeholders, the framework (Figure 2) outlines key components including remuneration rates, payment methods, non‐financial recognition approaches and decision‐making considerations.

### 4.1 Flexible Remuneration Rates

The framework proposes three remuneration rate options: project‐based rates (lump sums based on project scope), meeting rates ($200 per meeting lasting up to 4 h) and hourly rates ($50/h). This approach addresses two key considerations.

First, adopting a single payment rate rather than offering multiple options could limit the framework's adaptability across diverse research contexts. While Fox et al.'s 2023 scoping review informed this study's evidence base by proposing remuneration values, it was limited to three engagement levels and did not account for contextual variability [13]. Flexible payment options can better address consumers' differing needs and circumstances.

Second, although the review identified substantial variability in recommended payment amounts, findings from our survey and workshops (Table 5) showed minimal variation in current practices across engagement levels [13]. This suggests a relative consistency in remuneration approaches across Australia, reflecting a shared understanding of valuing consumer contribution within the research community. This alignment between current practices and the values expressed throughout our research indicates an encouraging level of consensus among Australian researchers regarding appropriate consumer recognition. Furthermore, most health consumer organisations (Table S1) prefer hourly or flat rates, which align with workshop discussions (Table 5), where few participants supported differing rates by engagement level. By offering flexible payment options, the framework aligns with literature advocating for diverse remuneration models to accommodate varying consumer needs and circumstances [2].

### 4.2 Diverse Payment Methods

The proposed framework includes diverse payment methods such as gift cards, bank transfers (honoraria), prepaid Visa or MasterCard, cash, employment of consumers and other options such as donations to a designated charity. This flexibility allows consumers to choose options suited to their individual circumstances. This aligns with previous research, which highlighted that some consumers decline remuneration or opt out entirely due to concerns such as tax implications or pension impacts [27]. Accordingly, we recommend presenting all available options to consumers to address diverse needs.

### 4.3 Reimbursement Considerations

The framework does not explicitly separate reimbursement from remuneration, as findings from deliberative workshops (Table 6) indicated that all out‐of‐pocket costs should be covered by remuneration. Nevertheless, we recommend informing consumers about the distinction between remuneration and reimbursement, as well as the specific scope of reimbursement, at the beginning of their engagement. This clarity is essential for effective recognition of their contributions.

One possible reason for this lack of distinction is the absence of national remuneration guidelines in Australia, which may result in limited understanding of remuneration terminology among research organisations and consumers. Communicating the reimbursement scope that the project can cover may help reduce barriers to engagement, fostering inclusivity in consumer engagement.

### 4.4 Non‐Financial Recognition Approaches

Similar to remuneration methods, the framework offers various non‐financial recognition approaches, including co‐authorship or acknowledgement on academic outputs, co‐presentations at conferences, skills development opportunities, team building and other options (e.g., public recognition or mentorship). This range accommodates individual preferences, with the ‘other’ option added based on deliberative workshop suggestions (Table 7).

While co‐authorship or acknowledgement is commonly reported in the literature, it is essential that consumers choosing this option understand authorship criteria, which for consumers usually differ from the requirements for academics [2]. The International Committee of Medical Journal Editors (ICMJE) authorship criteria are very clear for academics, although no criteria have been developed specifically for consumers. Research indicates that around 69% of Editors‐in‐Chief support consumer partners as co‐authors in biomedical research, yet there is ongoing debate about whether the ICMJE criteria should be revised to better reflect consumer contributions [28]. In practice, most academics working with consumers set co‐authorship criteria based on their own judgement, as only about 4% of journals have formal policies addressing consumer authorship [28]. This is a gap that needs to be addressed to foster consumer co‐authorship in health research. Recent initiatives, such as the introduction of the ‘Patient Author’ metatag in publication affiliations, show promise, resulting in a nine‐fold increase in identifiable patient‐authored articles between 2020 and 2021 [29].

Although non‐financial recognition is more frequently reported, it cannot replace remuneration. Working group members emphasised the importance of providing remuneration whenever possible to appropriately value consumers' contributions to research.

### 4.5 Guided Decision‐Making Process

The inclusion of four questions to guide decision‐making in the framework emphasises the importance of early preparation [2, 27, 30]. Researchers should determine available remuneration options and reimbursement coverage at the project's outset and share this information during initial discussions with consumers, aligning with literature that highlights the need to address logistical considerations in advance [30].

Researchers should then engage consumers in discussions about their preferred recognition forms, considering individual circumstances and financial implications, including concerns about disclosing personal information, such as home address or social insurance number, when receiving remuneration [13]. By enabling consumers to make informed decisions, this framework fosters mutual trust and facilitates the development of a tailored remuneration strategy, balancing recognition, autonomy and the potential impacts of financial payments while helping consumers establish clear expectations regarding their roles and remuneration.

### 4.6 Limitations and Future Directions

While our framework offers a tool for acknowledging consumers' contributions to research, several limitations must be noted. Due to the anonymous nature of the survey, we could not determine whether multiple responses came from the same organisation. As such, we cannot assess possible over‐representation or confirm whether responses reflected distinct organisational perspectives.

Recruitment for the survey was limited to health organisations affiliated with Research Australia, which may not reflect the full diversity of health research organisations in Australia. However, workshop participants were drawn from both the research team and Research Australia's networks and included representatives from a range of organisations and consumers with various health conditions (e.g., diabetes, rare diseases, cancer, dementia and neurodegenerative diseases). Nonetheless, people from marginalised groups, such as Indigenous Australians, overseas‐born individuals or non‐native English speakers, were not represented in the workshops, limiting the broader applicability of the study's findings.

Although only 21 participants attended the six deliberative workshops, the small group format enabled in‐depth discussion of the draft frameworks, which had been developed using peer‐reviewed literature, state‐ and territory‐based evidence, and survey findings. Group size was constrained by participants' availability. Workshop insights were further discussed with the project working group, contributing to the refinement and confirmation of the final framework.

The project working group comprised member organisations of Research Australia and may not represent the wider research and consumer engagement landscape. We encourage researchers and institutions applying this framework to report their remuneration practices transparently, and we recommend further evaluation and validation in different research contexts, including funded and unfunded institutions and for‐profit and not‐for‐profit organisations, to support ongoing validation and refinement.

## 5 Conclusion

This study developed the first framework to guide the Australian research community in recognising consumers' contributions to health research. The framework integrates international best practices, relevant state and territory policies, and comprehensive stakeholder insights. It outlines four key components for recognising contributions: decision‐making considerations, remuneration rates, payment methods and non‐financial recognition approaches.

While this framework is not the only model for recognition decision‐making, it serves as a valuable starting point for dialogue on this critical topic and to inform future policy development. Further evaluation, incorporating the experiences and perspectives of consumer partners across diverse research contexts, is essential to enhance the framework's applicability and to foster fair and consistent recognition for valuable contributions to health research.

## Author Contributions

**Mingming Zhou:** conceptualisation, methodology, data curation, formal analysis, validation, investigation, visualisation, project administration, writing – review and editing, writing – original draft, software. **Anne Parkinson:** supervision, methodology, data curation, investigation, validation, formal analysis, writing – review and editing. **Lucy Clynes:** conceptualisation, data curation, writing – review and editing, resources. **Jane Desborough:** conceptualisation, methodology, data curation, supervision, resources, validation, investigation, formal analysis, writing – review and editing.

## Ethics Statement

This study received ethics approval from the Australian National University Human Research Ethics Committee (Protocol 2023/1263).

## Consent

All participants provided informed consent.

## Conflicts of Interest

The authors declare no conflicts of interest.

## Supporting information

R2 Supporting Figures 1 2.

R2 Supporting Tables 1 5.

## Data Availability

The data that support the findings of this study may be available from the corresponding authors upon reasonable request.
