# Supplementary material for: Recognising Consumers' Contributions to Health Research: Co‐Designing a Remuneration Framework for the Australian Context
Source: Health Expect. 2025 May 28;28(3):e70314. doi: 10.1111/hex.70314 (PMC12119376; doi:10.1111/hex.70314)
Supplement: Supplementary file 1 — R2 Supporting Figures 1 2. [file HEX-28-e70314-s002.docx]

**Supplementary Figures:**

**
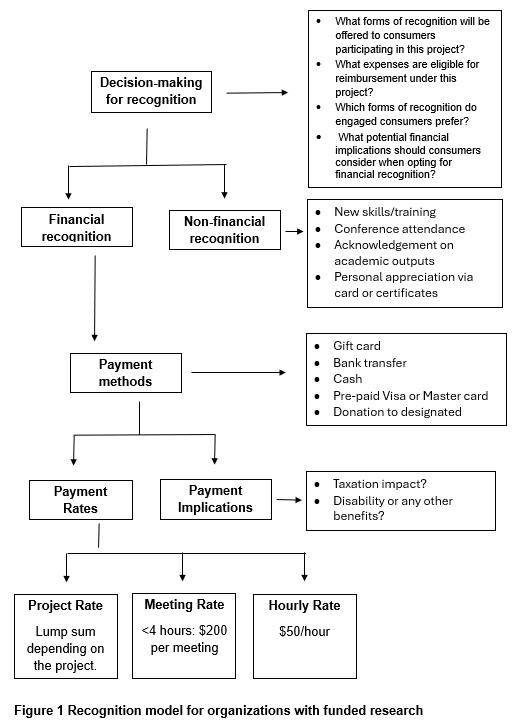
**

**Figure 1 Recognition model for organisations with funded research**


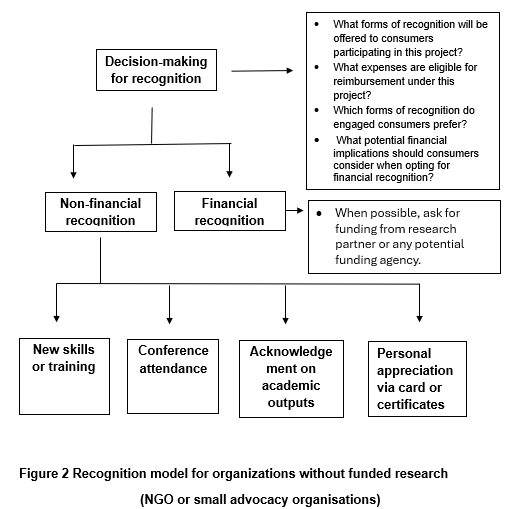


**Figure 2 Recognition model for organisations without funded research (NGO or small advocacy organisations)**
