# Supplementary material for: Recognising Consumers' Contributions to Health Research: Co‐Designing a Remuneration Framework for the Australian Context
Source: Health Expect. 2025 May 28;28(3):e70314. doi: 10.1111/hex.70314 (PMC12119376; doi:10.1111/hex.70314)
Supplement: Supplementary file 2 — R2 Supporting Tables 1 5. [file HEX-28-e70314-s001.docx]

**Supplementary Table 1 Remuneration methods recommended by state-based consumer organisations in Australia***

|  | Organization | Release date | Remuneration methods | |
| --- | --- | --- | --- | --- |
| NSW | Health Consumers NSW | Oct 2022 | Hourly rate based on level of engagement | Empower/lead the research, Collaborate/Partner –Strategic Leadership role: $60 per hour; Collaborate/Partner/Advise: $50 per hour; Consult: $40 per hour; Inform: no payment. |
| QLD | Health Consumers Queensland | July 2024 review | Flat rate based on level of engagement | Consumer representatives engaged for ongoing, regular, high-level committees: $234 per meeting 4 hours and under, $469 per meeting over 4 hours. (These two rates incorporate pre-reading and travel time); Consumers engaged in operational level committees and meetings or less formal ways: $50 per hour. |
| ACT | Health Care Consumers Association | July 2024 review | Flat rate | Follows ACT Health guidelines: $75.00 for the initial two hours + $18.75 foreach additional 15 minutes. |
| WA | WA Health Consumers’ council | Jan 2024 | Hourly rate | $37.50 per hour or part thereof (2 hour minimum) for the following activities: committee membership (executive, boards, steering, advisory, reference groups); working parties; forums, focus groups and workshops; selection, evaluation, and recruitment panels. |
| VIC | Health Consumers Centre | March 2023 | Both flat rate and hourly rate based on level of engagement | Victorian Comprehensive Cancer Centre (VCCC) alliance: Sitting fee for consumer-led/partnership level of engagement: Committee Chair: $238 (≥ 4h); $119 (< 4h); Committee Member: $206 (≥4 h); $103 (< 4h). Sitting fee for Involving: $50/h; Sitting fee for Consulting: $40/h. |
| TAS | Health Consumers Tasmanian | Sep 2022 | Flat rate | Not found. TAS Health Senate: Consumer, Carer, Peak Health Organisations and Community Representatives: $100 per meeting up to 3 hours; $175 per meeting 3-5 hours; $250 per meeting >5 hours. |
| SA | Health Consumer CoLab | June 2021 | Hourly rate | Consumer Sitting Fees and Reimbursement: $45/hour sitting fee or set time duration rate that is in proportion to the level of skills, knowledge and contribution as an equal and expert partner in decision-making with the other experts in their field. |
| NT | No Health Consumers organisation identified |  |  | - |

*****Retrieval date: August 2, 2024

**Supplementary Table 2 Remuneration methods from survey results**

| **Level of engagement** | **Survey results** | | |
| --- | --- | --- | --- |
|  | n | % | Description |
| Consumer-leading | 20 | 6.7 | Consumer-led advocate |
| Collaboration | 72 | 24.4 | Team members |
| Active participation (Cooperation) | 109 | 36.4 | Participants, Other (document review, presentation delivery) |
| Consultation | 73 | 24.4 | Advisory group or steering committee, Involvement coordinators |
| Informing | - | - | No results for this level |

**Supplementary Table 3 Model 1: remuneration rates**

|  |  | **Consultation** | **Collaboration** | **Active Participation (Cooperation)** |
| --- | --- | --- | --- | --- |
| Rates from  (Fox, 2024)^#^ | Rate  Range | 29 AUD/hour^##^  Range: 18 ~ 75 AUD/hour | 57 AUD/hour  Range: 38~ 75 AUD/hour | 86 ~ 520 AUD for document reviewer  114 AUD preparing and delivering a formal presentation  171~ 260 AUD active participation in an external event or attending training  129 ~ 137 AUD as a patient partner interviewer  228 AUD per conference attended |
|  |  | 571 ~ 915 AUD/year | 1144 ~ 1715 AUD/year |  |
| Rates from  state-based consumer organisations* |  | NSW: AUD $40/h.  QLD: AUD $ 234 per meeting 4 hours and under, AUD $ 469 per meeting over 4 hours.  WA: AUD $37.50 per hour (2 hour minimum).  VIC: AUD $40/h. | NSW: AUD $50/h.  QLD: AUD $ 50/h.  VIC: AUD $50/h.  SA: AUD $ 45/h | ACT: flat rate, AUD $75.00 for the initial two hours + $18.75 foreach additional 15 minutes.  TAS: flat rate, AUD $100 per meeting up to 3 hours; AUD $175 per meeting 3-5 hours; AUD $250 per meeting >5 hours. |
| Rates from RA survey results | Min-Max | 35~ 100 AUD/hour  50 ~ 400 AUD/meeting | 20 ~ 100 AUD/hour  80 ~ 750 AUD/meeting | 23 ~ 50 AUD/hour  5 ~ 100 AUD/meeting |
|  | Interquartile range (25-75%) | 35 ~ 50 AUD/hour  100 ~ 200 AUD/meeting | 30 ~ 50 AUD/hour  200 ~ 300 AUD/meeting | 23 ~ 50 AUD/hour  20 ~ 50 AUD/meeting |

^#^Fox, Grace, et al. "What guidance exists to support patient partner compensation practices? A scoping review of available policies and guidelines." *Health Expectations* 27.1 (2024): e13970.

^##^The transferred currency rate was 1USD = 1.503 AUD (June 1, 2024).

*Supplementary Table 1 Remuneration methods recommended by state-based consumer organisations in Australia

**Supplementary Table 4 Model 2: Remuneration methods and reimbursement coverage**

|  | **Payment methods** | | | **Reimbursement coverage** | | |
| --- | --- | --- | --- | --- | --- | --- |
|  |  | n* | % |  | n | % |
| Methods from  (Fox, 2024) ** | Honoraria | 43 | 69 | Conference attendance | 25 | 43 |
|  | Gift card | 11 | 18 | Babysitting/caregiver services | 21 | 36 |
|  | Salary | 9 | 15 | Accommodation | 9 | 16 |
|  | Stipend | 4 | 6 |  |  |  |
|  |  | N^#^ | % |  | N | **%** |
| Methods from  RA survey results | Cash | 11 | 6.2 | Transport (taxi, bus) | 49 | 33.6 |
|  | Bank account transfer | 54 | 30.3 | Parking fees | 46 | 31.5 |
|  | Gift card | 71 | 39.9 | Time for pre-reading | 39 | 26.7 |
|  | Pre-paid Visa or Master card | 32 | 18 | Something not listed above | 12 | 8.2 |
|  | Similar as employees | 10 | 5.6 |  |  |  |

n* referred to the number of guidance and policy documents identified in the review.

N^#^ referred to the number of participants responded to the survey.

**Fox, Grace, et al. "What guidance exists to support patient partner compensation practices? A scoping review of available policies and guidelines." *Health Expectations* 27.1 (2024): e13970.

**Supplementary Table 5 Model 3: Non-financial recognition methods**

| Methods from the latest review  (Fox, 2024) | | | Methods from survey results | | |
| --- | --- | --- | --- | --- | --- |
|  | n* | % |  | N# | % |
| Services or training opportunities | 26 | 40 | Provision of training or opportunities for skills development | 37 | 22.7 |
| Acknowledgement on research outputs | 10 | 15 | Academic recognition, such as co-authorship or acknowledgment on publications | 46 | 28.2 |
| Conference presentation opportunities | 25 | 38 | Co-presentations at conferences | 33 | 20.2 |
| Invitation to a special event | 3 | 4 | Team building activities, such as social gatherings | 36 | 22.1 |
| Co-authorship | 6 | 9 | Other not listed above^##^ | 11 | 6.7 |
| Meal provision | 11 | 17 |  |  |  |
| Verbal thank you | 9 | 14 |  |  |  |
| In-kind donation | 9 | 14 |  |  |  |
| Gift | 10 | 15 |  |  |  |
| Gift cards^a^ | 9 | 14 |  |  |  |
| Honorary appointment | 3 | 4 |  |  |  |
| Coinvestigator/co-applicant on grant | 1 | 1 |  |  |  |

n* referred to the number of guidance and policy documents identified in the review.

N# referred to the number of participants responded to the survey.

^##^ Free-text responses for this choice ‘other not listed about’ included: contributions for community, mentorship or consultation, peer-support, results sharing, student credits and gifts.

^a^ Gift card offered as a token of appreciation, which is different from the gift cards (for grocery stores, restaurants, retail stores, prepaid visa gift cards etc.) that are considered financial compensation when the value is informed by a formal conversion or consumer partners decide that they want to receive payment in the form of gifts or gift cards.

Fox, Grace, et al. "What guidance exists to support patient partner compensation practices? A scoping review of available policies and guidelines." *Health Expectations* 27.1 (2024): e13970.
